# Supplementary material for: Motility-induced coexistence of a hot liquid and a cold gas
Source: Nat Commun. 2024 Apr 13;15:3206. doi: 10.1038/s41467-024-47533-9 (PMC11016108; doi:10.1038/s41467-024-47533-9)
Supplement: Supplementary file 1 — Supplementary Information [file 41467_2024_47533_MOESM1_ESM.pdf]

Supplementary Information: Motility-induced coexistence of a hot liquid and a cold gas

Lukas Hecht,[1] Iris Dong,[1] and Benno Liebchen[1]

[1] *Institute of Condensed Matter Physics, Department of Physics, Technical University of Darmstadt, Hochschulstraße 8, 64289 Darmstadt, Germany*

Violation of the equipartition theorem

The different persisting temperatures are accompanied by a violation of the equipartition theorem, which holds for classical systems in equilibrium. It states that each degree of freedom [which is quadratic in the (momentum) coordinates] contributes (on average) with $k_\mathrm{B}T/2$ to the total energy of the system [1, 2]. This would imply that the kinetic temperature of the active and passive particles are the same, which is in fact the case for a completely overdamped system (Fig. S9). Even for the case of overdamped active and underdamped passive particles, the equipartition theorem applies for small Pe, where the dynamics of the system is dominated by thermal diffusion and the system is near equilibrium (Figs. S10 and S11a,b). However, the ratio $T_\mathrm{kin}^\mathrm{passive}/T_\mathrm{kin}^\mathrm{active}$ of the kinetic temperature of the passive and active particles increases significantly with increasing Pe both in the uniform regime and in the coexistence regime (Figs. S10 and S11a,b). Note that the ratio $T_\mathrm{kin}^\mathrm{passive}/T_\mathrm{kin}^\mathrm{active}$ is largest at large x_a (and large Pe) in the dense phase (Figs. S10 and S11a,c), whereas in the dilute phase, it reaches its maximum at intermediate (small Pe) or small (large Pe) x_a (Fig. S11d), which is in line with our analysis leading to the transition between the scenarios hot-liquid–cold-gas and hot-gas–cold-liquid.

Effective forces on passive particles

At low and intermediate Pe, we have shown that the passive particles are colder in the dense phase compared to the dilute phase in terms of their kinetic temperature. As described in the main text, the mechanism of this phenomenon is based on a trapping of inertial passive particles within the dense phase. In contrast, in the dilute phase, active particles can push passive particles forward and persistently speed them up. One key ingredient of this mechanism is that passive particles remain trapped and are densely packed within the dense phase, which is not trivial to be the case for inertial passive particles. To explore this in more detail, we have calculated the effective force

acting on the passive particles depending on their position. To this end we made a simulation in a slit geometry in which the border of the dense phase is approximately stationary and does not move much, which allows to perform a long-time average. As shown in Fig. S12b, the effective force always points to the dense phase at its border, i.e., the passive particles are pushed inside the dense phase. This effective force finally ensures, that the passive particles remain densely packed within the dense phase.

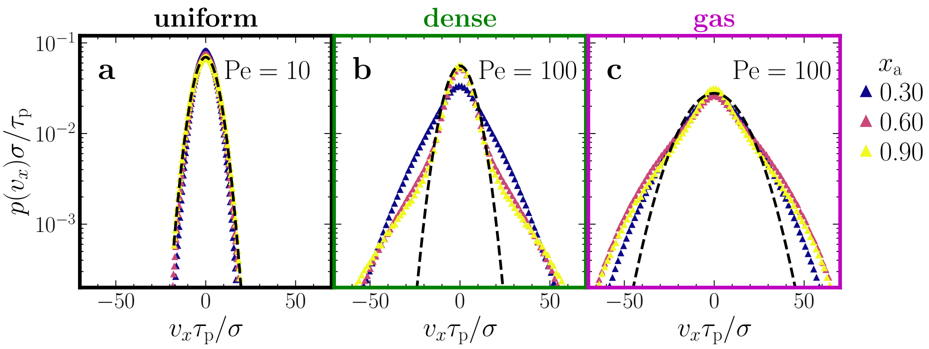

FIG. S1. **Velocity distribution.** Distribution of the x component of the velocities of the passive particles **a** in the uniform state at $\mathrm{Pe} = 10$ and in the MIPS state at $\mathrm{Pe} = 100$ **b** in the dense phase and **c** in the gas phase for different values of x_a as given in the key (other parameters as in Fig. 2 in the main text). The black dashed lines are Gaussian fits showing that the distributions are clearly non-Gaussian in the MIPS state.

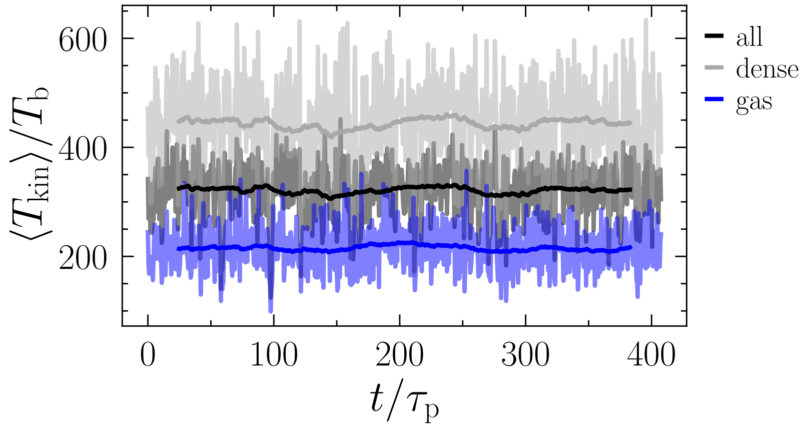

FIG. S2. **Kinetic temperature over time.** Kinetic temperature of the passive particles in the MIPS state over time averaged over all particles (black), particles in the dense phase (gray), and particles in the dilute phase (blue). The darker lines are moving averages. Parameters: $\mathrm{Pe} = 400$, $x_\mathrm{a} = 0.9$ (other parameters as in Fig. 2 in the main text).

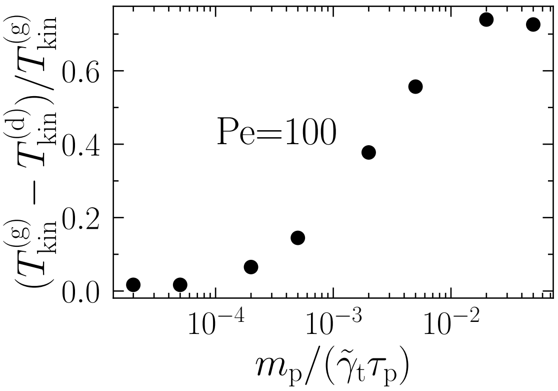

FIG. S3. **Kinetic temperature difference.** Normalized difference of the kinetic temperature of passive particles in the dense and in the gas phase as function of the mass m_p of the passive particles. Parameters: $x_a = 0.6$, $\text{Pe} = 100$, $m_a/(\gamma_t \tau_p) = 5 \times 10^{-5}$ (other parameters as in Fig. 2 in the main text).

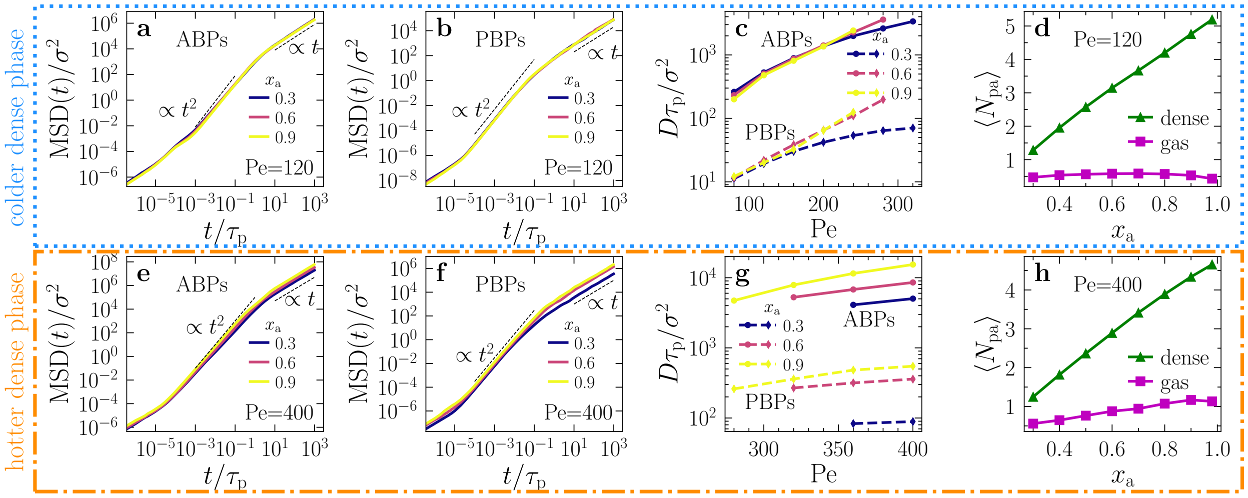

FIG. S4. **Diffusive dynamics and number of active neighbors. a,b** Mean square displacement (MSD) of the active and passive particles, respectively, for $\text{Pe} = 120$ and three values of x_a as shown in the key. **c** Long-time diffusion coefficients of the ABPs (solid lines) and PBPs (dashed lines) in region (II_a) of the phase diagram (cf. Fig. 6 in the main text). **e,f** MSD of the active and passive particles, respectively, for $\text{Pe} = 400$ and three values of x_a given in the key. **g** Long-time diffusion coefficients of the ABPs (solid lines) and PBPs (dashed lines) in region (II_b) of the phase diagram (cf. Fig. 6 in the main text). **d,h** Mean number of active particles in contact with passive particles (i.e., with a distance smaller than the cutoff distance r_c of the WCA potential) for $\text{Pe} = 120$ and $\text{Pe} = 400$, respectively. All other parameters are the same as in Fig. 2 in the main text.

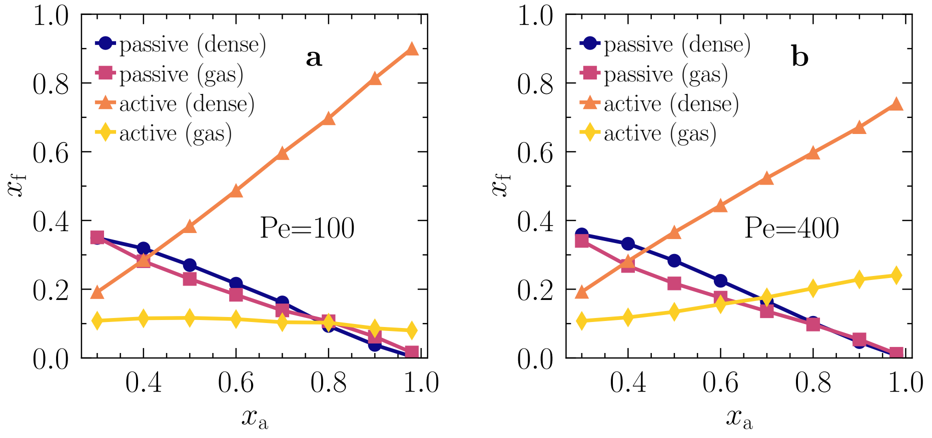

FIG. S5. **Fraction of particles in dense and dilute phases.** Fraction $x_\mathrm{f} = N/(N_\mathrm{a} + N_\mathrm{p})$, where N denotes the number of active or passive particles in the dense or dilute phase **a** at Pe $= 100$ and **b** at Pe $= 400$ (other parameters as in Fig. 2 in the main text).

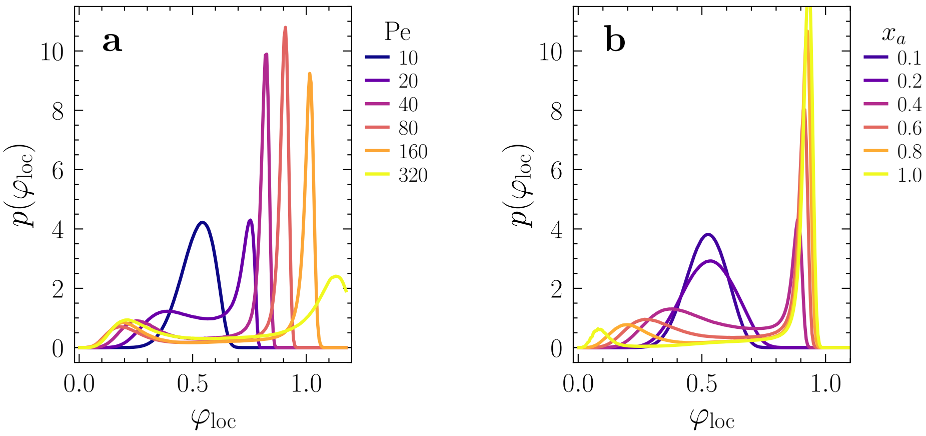

FIG. S6. **Local area fraction.** **a** Distribution of the local area fraction φ_loc at $x_\mathrm{a} = 0.8$ for different Pe values as given in the key. **b** Distribution of φ_loc at Pe $= 80$ for different x_a values as given in the key (other parameters as in Fig. 2 in the main text). We calculated $p(\varphi_\mathrm{loc})$ via averages over circles of radius 5σ and over time in the steady state.

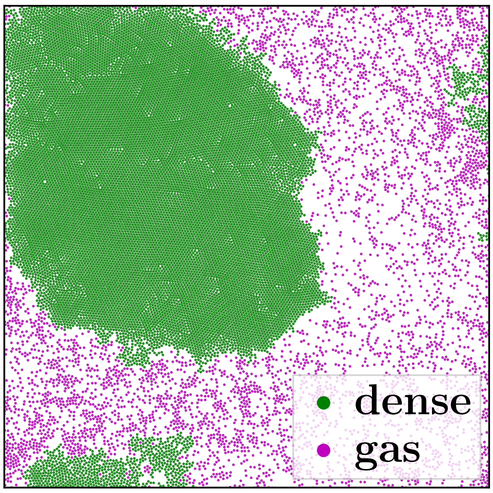

FIG. S7. **Distinction between dense and dilute phase.** Exemplary snapshot demonstrating the distinction between particles in the dense and the dilute phase obtained from the identification of the largest cluster. Here, a cluster is defined based on the distance between the particles such that two particles belong to the same cluster if their distance to each other is smaller than the cutoff distance $r_\mathrm{c} = 2^{1/6}\sigma$ of the repulsive pairwise interaction potential. Parameters: Pe $= 100$, $x_\mathrm{a} = 0.6$ (other parameters as in Fig. 2 in the main text).

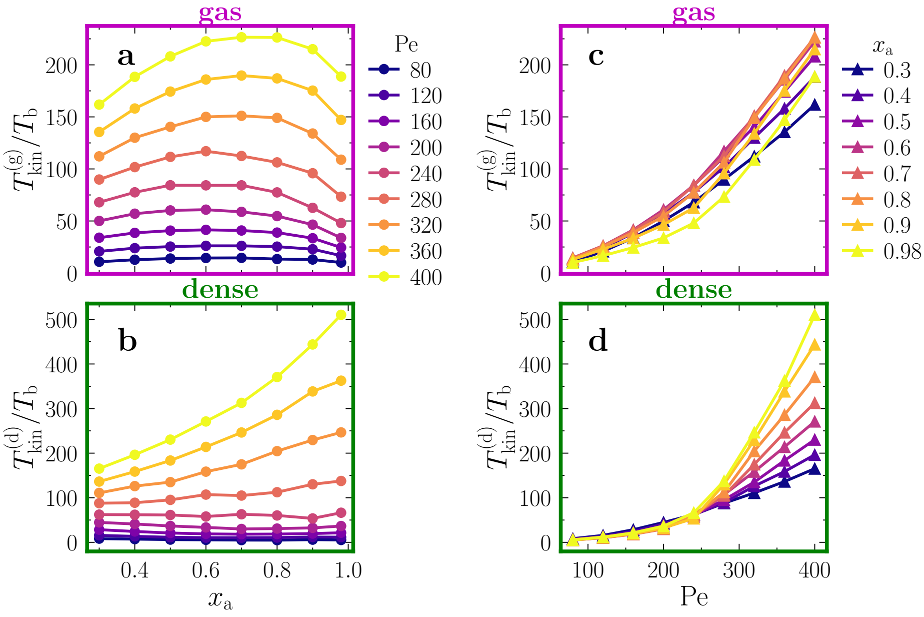

FIG. S8. **Kinetic temperature.** Kinetic temperature of the passive particles **a,c** in the dilute phase and **b,d** in the dense phase in the MIPS state for different values of Pe and x_a as given in the key. Parameters as in Fig. 2 in the main text.

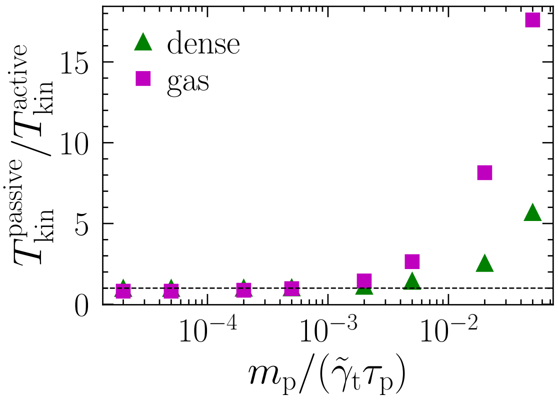

FIG. S9. **Mass dependence of the violation of the equipartition theorem.** Ratio between the kinetic temperature of the active and the passive particles in the dense phase (triangles) and the gas phase (squares) as function of the mass $m_{\rm p}$ of the passive particles. Parameters: $\mathrm{Pe} = 100$, $x_{\rm a} = 0.6$, and $m_{\rm a}/(\gamma_{\rm t}\tau_{\rm p}) = 5 \times 10^{-5}$ (other parameters as in Fig. 2 in the main text).

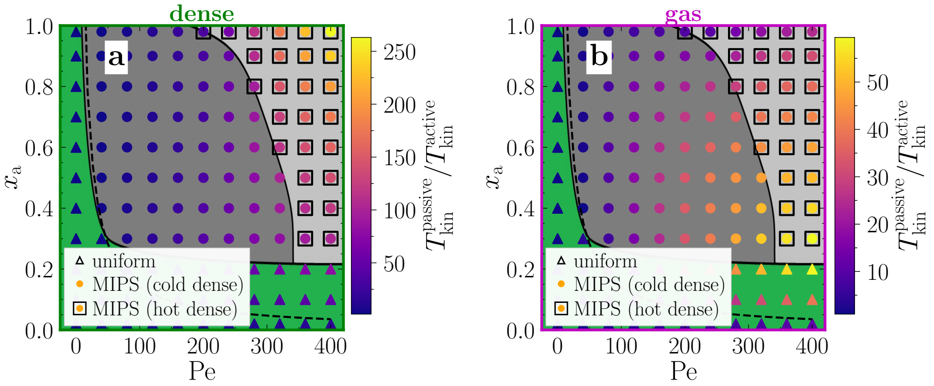

FIG. S10. **Violation of the equipartition theorem.** Same non-equilibrium phase diagram as in Fig. 6 in the main text but the colors now denote the ratio of the kinetic temperature of the passive particles and the active particles **a** in the dense phase and **b** in the gas phase with values given in the key.

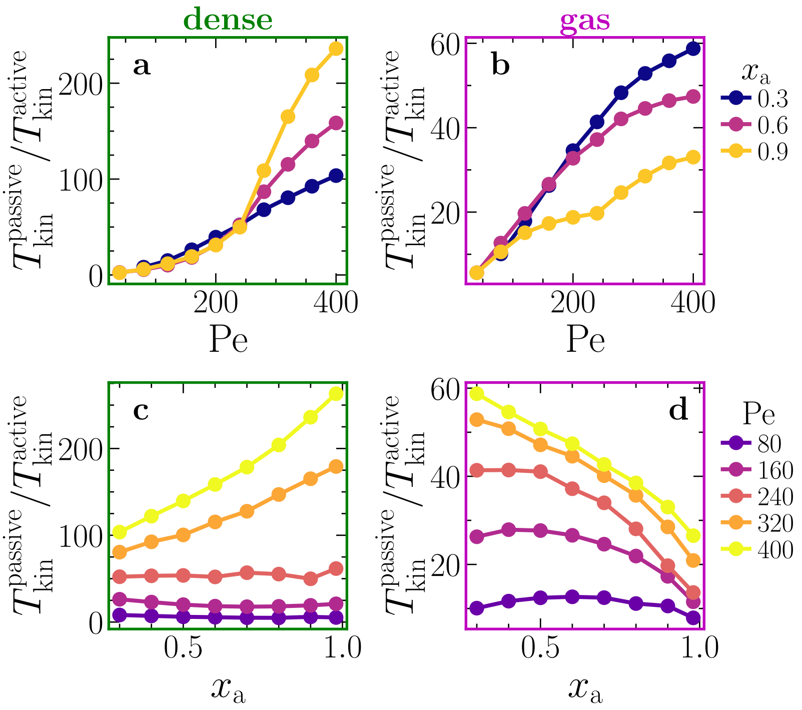

FIG. S11. **Kinetic temperature ratios.** Cuts through the non-equilibrium phase diagram in Fig. S10 showing the ratio of the kinetic temperature of the passive particles and the active particles **a**,**b** as function of Pe for three different values of $x_{\rm a}$ given in the key and **c**,**d** as function of $x_{\rm a}$ for different Pe (values are given in the key) in the dense and gas phase, respectively (other parameters as in Fig. 2 in the main text).

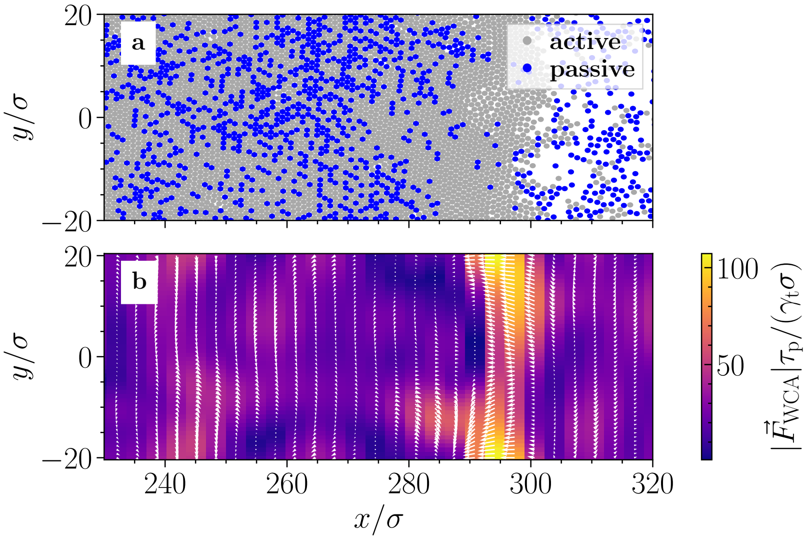

FIG. S12. **Effective force on passive particles. a** Snapshot of the binary mixture in a thin rectangular box showing motility-induced phase separation. The box shape ensures that the border of the dense phase is approximately stationary allowing for long-time averages. Here, we show an extract from the simulation showing one interface and a part of the dense and the dilute phase in its vicinity. **b** Corresponding coarse-grained force field of the interaction force from the Weeks-Chandler-Anderson (WCA) potential acting on the passive particles. The color and arrow length represent the strength of the effective force, the orientation of the white arrows its direction. A strong effective force is pushing passive particles towards the dense phase (yellow region). Parameters: $\mathrm{Pe} = 100$, $x_{\mathrm{a}} = 0.6$, $L_x = 784\sigma$, $L_y = 40\sigma$ (other parameters as in Fig. 2 in the main text).

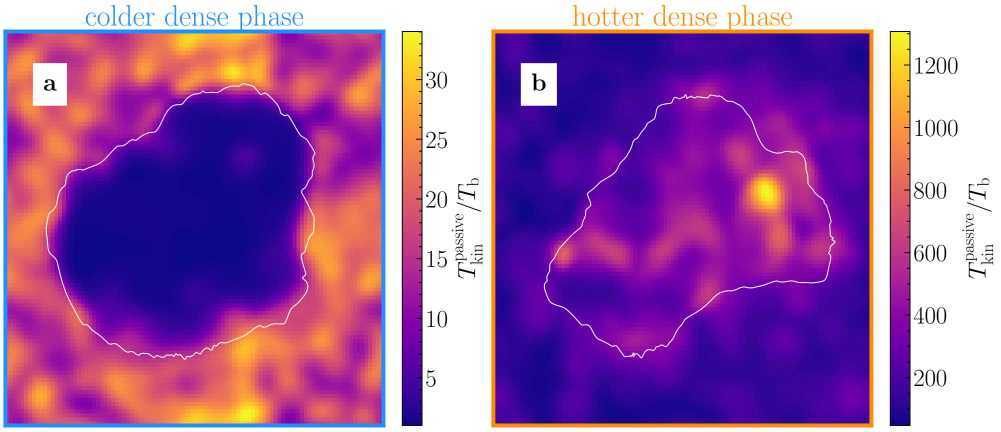

FIG. S13. **Zero-inertia limit for active particles.** To show that our results persist even when considering zero inertia for the active particles, we also made simulations using the overdamped Langevin equation for the active particles for the scenario **a** cold-liquid–hot-gas and the opposite scenario **b** hot-liquid–cold-gas. Here, we show the corresponding coarse-grained kinetic temperature fields. The white lines denote the border of the dense phase, which is located inside the area enclosed by the white lines. Parameters used in panels a and b are the same as in Fig. 1e–h and i–l in the main text, respectively, but with $m_{\rm a}/(\gamma_{\rm t}\tau_{\rm p}) = 0$ and $I/(\gamma_{\rm r}\tau_{\rm p}) = 0$.

TABLE S1. **Temperature definitions.** Exemplary temperature values of the passive particles in the dense and the gas phase obtained from the different temperature definitions. The values for (A) and (B) correspond to the two simulations shown in Fig. 8a–c and d–f in the main text, respectively. For the latter, the velocity distribution is not Gaussian, and therefore, T_{MB} cannot be determined.

		$T_{\mathrm{kin}}/T_{\mathrm{bath}}$	$T_{\mathrm{kin,rel}}/T_{\mathrm{bath}}$	$T_{\mathrm{MB}}/T_{\mathrm{bath}}$
**A** (Fig. 8a–c)	dense	4.4×10^2	2.8×10^2	4.3×10^2
	gas	2.2×10^2	2.2×10^2	1.7×10^2
**B** (Fig. 8d–f)	dense	6.6×10^1	6.2×10^1	-
	gas	5.0×10^1	4.9×10^1	-

[1] P. A. Mello and R. F. Rodríguez, The equipartition theorem revisited, Am. J. Phys. **78**, 820 (2010).
[2] D. V. Schroeder, *An Introduction to Thermal Physics* (Oxford University Press, Oxford, 2021).
