# Supplementary material for: Motility-induced coexistence of a hot liquid and a cold gas
Source: Nat Commun. 2024 Apr 13;15:3206. doi: 10.1038/s41467-024-47533-9 (PMC11016108; doi:10.1038/s41467-024-47533-9)
Supplement: Supplementary file 3 — Description of Additional Supplementary Files [file 41467_2024_47533_MOESM3_ESM.pdf]

Description of Additional Supplementary Files

File name: Supplementary Movie 1

Description: Snapshot (left) and coarse-grained kinetic temperature field of the passive tracer particles (right) of a mixture of overdamped active Brownian particles with overdamped passive tracers (as in Fig. 1a–d in the main text). Parameters: $x_{\mathrm{a}} = 0.6$, $\mathrm{Pe} = 100$, $m_{\mathrm{a}}/(\gamma_{\mathrm{t}}\tau_{\mathrm{p}}) = 5 \times 10^{-5}$, $m_{\mathrm{p}}/(\tilde{\gamma}_{\mathrm{t}}\tau_{\mathrm{p}}) = 5 \times 10^{-5}$, $N_{\mathrm{a}} + N_{\mathrm{p}} = 20\,000$, $\varphi_{\mathrm{tot}} = 0.5$.

File name: Supplementary Movie 2

Description: Snapshot (left) and coarse-grained kinetic temperature field of the passive tracer particles (right) of a mixture of overdamped active Brownian particles with inertial passive tracers showing coexistence of a hot gas-like and a cold liquid-like phase (as in Fig. 1e–h in the main text). Parameters: $x_{\mathrm{a}} = 0.6$, $\mathrm{Pe} = 100$, $m_{\mathrm{a}}/(\gamma_{\mathrm{t}}\tau_{\mathrm{p}}) = 5 \times 10^{-5}$, $m_{\mathrm{p}}/(\tilde{\gamma}_{\mathrm{t}}\tau_{\mathrm{p}}) = 5 \times 10^{-2}$, $N_{\mathrm{a}} + N_{\mathrm{p}} = 20\,000$, $\varphi_{\mathrm{tot}} = 0.5$.

File name: Supplementary Movie 3

Description: Snapshot (left) and coarse-grained kinetic temperature field of the passive tracer particles (right) of a mixture of overdamped active Brownian particles with inertial passive tracers showing coexistence of a cold gas and a hot liquid-like droplet (as in Fig. 1i–l in the main text). Parameters: $x_{\mathrm{a}} = 0.9$, $\mathrm{Pe} = 400$, $m_{\mathrm{a}}/(\gamma_{\mathrm{t}}\tau_{\mathrm{p}}) = 5 \times 10^{-5}$, $m_{\mathrm{p}}/(\tilde{\gamma}_{\mathrm{t}}\tau_{\mathrm{p}}) = 5 \times 10^{-2}$, $N_{\mathrm{a}} + N_{\mathrm{p}} = 20\,000$, $\varphi_{\mathrm{tot}} = 0.5$.

File name: Supplementary Movie 4

Description: Simulation of a mixture of overdamped active Brownian particles with inertial passive tracers showing coexistence of a cold gas and a hot liquid-like droplet (as in Fig. 1i–l in the main text). An exemplary trajectory of a passive tracer particle is marked in red demonstrating how a passive tracer particle is pushed forward in the dense phase as a result of correlated dynamics of the active particles. The right panel shows a zoomed version of the left panel. Parameters: $x_{\mathrm{a}} = 0.9$, $\mathrm{Pe} = 400$, $m_{\mathrm{a}}/(\gamma_{\mathrm{t}}\tau_{\mathrm{p}}) = 5 \times 10^{-5}$, $m_{\mathrm{p}}/(\tilde{\gamma}_{\mathrm{t}}\tau_{\mathrm{p}}) = 5 \times 10^{-2}$, $N_{\mathrm{a}} + N_{\mathrm{p}} = 20\,000$, $\varphi_{\mathrm{tot}} = 0.5$.
